# Differentiating the Neuropharmacological Properties of Nicotinic Acetylcholine Receptor-Activating Alkaloids

**DOI:** 10.3389/fphar.2022.668065

**Published:** 2022-03-22

**Authors:** Omar Alijevic, Oihane Jaka, Ainhoa Alzualde, Diana Maradze, Wenhao Xia, Stefan Frentzel, Andrew N. Gifford, Manuel C. Peitsch, Julia Hoeng, Kyoko Koshibu

**Affiliations:** ^1^ PMI R&D, Philip Morris Products S.A., Neuchâtel, Switzerland; ^2^ Biobide, Donostia-San Sebastian, Spain; ^3^ Gifford Bioscience Ltd., The BioHub Birmingham, Birmingham, United Kingdom; ^4^ PMI R&D, Philip Morris International Research Laboratories Pte. Ltd., Singapore, Singapore

**Keywords:** nicotine, cotinine, anatabine, alkaloids, nicotinic acetylcholine receptor (nAChR), anxiety

## Abstract

Alkaloids that target nicotinic acetylcholine receptors (nAChR) are of great interest because of the critical role they play in mood and anxiety. However, understanding of the neuropharmacological effects of nicotinic alkaloids, such as cotinine and anatabine, is very limited. In this study, we investigated the neuropharmacological effects of three naturally occurring alkaloids—nicotine, cotinine, and anatabine—*in vitro* and *in vivo*. A single injection of nicotine induced anxiolytic-like behavioral features in mice by using the SmartCube^®^ behavioral profiling system, while cotinine and anatabine had no detectable effect. The results were corroborated by using the zebrafish novel tank test (NTT), which showed a profound anxiolytic-like effect induced by multiple doses of nicotine after a single 20-min treatment. When the regulation of dopamine and norepinephrine release—the neurotransmitter systems relevant for anxiety—were examined *in vitro*, we found that nicotine stimulated the release of both norepinephrine and dopamine, while cotinine and anatabine mainly stimulated the dopamine release. The molecular targets of nicotine were confirmed to be nAChRs with its most potent activities against α4β2 and α6/3β2β3 subtypes *in vitro*. Anatabine was a weaker agonist for these receptors than nicotine. Cotinine was the least potent nAChR compound, only being able to activate α4β2 and α6/3β2β3 subtypes at high doses and no detectable activities against α3β4 and α7 subtypes at the concentrations tested. The observed effects were unlikely due to the off-target effect, because these alkaloids did not bind or regulate >160 other molecular targets *in vitro*. Thus, the present results suggest that natural nicotinic alkaloids can induce an anxiolytic-like behavior in nonclinical animal models, potency of which may depend on the activation of various nAChRs and regulation of various neurotransmitter systems. Further investigations would help understand their effects on humans, because non-clinical studies should not be taken as a direct indication for human behavior and nicotine is not risk free.

## 1 Introduction

Alkaloids are naturally occurring compounds present in a wide spectrum of plants, and their effects on animal behavior are being investigated for their therapeutic potential in various mood disorders and neurodegenerative diseases (Maione et al., 2013; Perviz et al., 2016; Hussain et al., 2018). There are more than 3,000 alkaloids identified, and their botanical and biochemical origins as well as chemical structures and pharmacological actions vary (Vina et al., 2012). In particular, pyridine alkaloids that target nicotinic acetylcholine receptors (nAChRs) are of great interest due to the critical role they play in neuropharmacology of mood and anxiety (Koob and Le Moal, 1997; Picciotto et al., 2002; Picciotto et al., 2015; Perviz et al., 2016). Nicotinic acetylcholine receptors are composed of *a* (α1–α10), *ß* (β1–β4), and other (δ, γ, ε) subunits, forming ligand-gated pentameric cation channels. Among the nAChRs, the homomeric α7 and heteromeric α4β2 nAChRs are the best characterized and most abundant subtypes in the central nervous system (Gotti et al., 2006). Other nAChRs in the brain can contain α3, α4, α5, α6, β2, β3, and β4 subunits in various combinations (Gotti and Clementi, 2004; Gotti et al., 2006). It is believed that the various receptor subtypes, inducing different time courses of activation and sensitization in various cell types involved in the diverse neurotransmitter systems, are responsible for the behavioral complexity induced by nicotinic compounds (Picciotto et al., 2002). For example, clinical studies suggest that abnormalities in cholinergic signaling are associated with major depressive disorder, whereas nonclinical studies have implicated both β2 subunit-containing (β2) and α7 nAChRs in anxiety- and depression-like behaviors (Perera et al., 2007; Mineur et al., 2013; Yu et al., 2014; Mineur et al., 2016). Thus, both nonclinical animal studies and clinical trials suggest that compounds that alter nAChR activity can affect behaviors related to mood and anxiety (Breslau, 1995; Diwan et al., 1998).

Among numerous alkaloids that activates nAChRs, nicotine is the most well-known natural alkaloid that can be found in many plants of the Solanaceae family with well-established activities on nAChRs (Alijevic et al., 2020; Xing et al., 2020). However, nicotine is not risk-free with reported negative effects on respiratory, gastrointestinal, cardiovascular functions and on addiction (Mishra, et al., 2015). A number of studies have also reported efficacy of nicotine in regulating memory, anxiety, and depression in rodents and humans (Levin, 2002; Terry et al., 2015; Bertrand and Terry, 2018; Terry and Callahan, 2019). In contrast, the effects of other alkaloids from the same chemical class in Solanaceae plans, such as cotinine and anatabine, are less well known (Dwoskin et al., 1995; Lippiello et al., 1996; Andersson et al., 2003; Vazquez-Palacios et al., 2004; Suemaru et al., 2006; Andreasen and Redrobe, 2009; Levin et al., 2014; Anderson and Brunzell, 2015; Terry et al., 2015; Xia et al., 2019). For example, anatabine is mainly known for its anti-inflammatory effect in neurodegenerative models in rodents (Paris et al., 2013a; Paris et al., 2013b; Verma et al., 2015), with a single study suggesting anxiolytic-like effect and improved social interaction and social memory in PS1/APPswe transgenic mice (Verma et al., 2015). In addition, little is known about the behavioral effects of anatabine when administered acutely.

In this study, the behavioral effects of three nicotinic alkaloids—nicotine, cotinine, and anatabine—were first assessed by using a proprietary machine learning system, SmartCube^®^, in order to discover their potential acute neurological effects in a relatively high-throughput manner. The SmartCube^®^ system allows phenotypic classification of test compounds by comparing the behavioral features induced by the compounds against a reference behavioral database built from known marketed drugs, including for example, buspirone, ipsapirone, and flesinoxan (Alexandrov et al., 2015; Alexandrov et al., 2016). The advantages of this system are automation of scoring and analysis and relatively high throughput, considering more than 2,000 of behavioral features obtained in one session. Using this innovative technology, the behavioral features induced by the three nicotinic alkaloids after a single intraperitoneal (i.p.) injection in mice were analyzed to understand their possible drug classifications. We chose to treat the animals acutely to understand the direct effect of the compounds on behavior without potential tolerability-related changes that are known to occur for nicotine (Perkins, 2002). In addition, the clinical references used to establish the behavior profile database for SmartCube^®^ used an acute single injection paradigm.

The three alkaloids were then examined by using the zebrafish novel tank test (NTT) of anxiety. The zebrafish NTT takes advantage of the innate behavior of zebrafish to dive and dwell at the bottom of a body of water when anxious. This behavioral paradigm is increasingly being accepted as a relative high-throughput method with some translational value to humans (Levin et al., 2007; Papke et al., 2012; Stewart et al., 2012). Nicotinic compounds as well as anxiolytic drugs, such as diazepam and buspirone, have been shown to induce anxiolytic-like effect in this zebrafish paradigm (Levin and Rezvani, 2007; Bencan and Levin, 2008; Bencan et al., 2009; Stewart et al., 2012). Lastly, the effects of these alkaloids on the neurotransmitter release and their molecular targets were assessed *in vitro* to understand the possible mechanisms underlying the behavioral findings.

## 2 Materials and Methods

### 2.1 Chemicals

(-)-Nicotine free base (CAS no. 54-11-5) and (-)-cotinine free base (CAS no. 486-56-6) were purchased from Sigma-Aldrich® (St. Louis, MO, United States). (±)-Anatabine citrate (purity 98.92% by HPLC) was purchased from Concept Life Sciences (Manchester, UK). (±)-Anatabine free base (purity >95% by HPLC) used for the SmartCube^®^ study was a generous gift from Indena® S. p.A. (Milan, Italy) (Rossia et al., 2018). (±)-Anatabine free base used for the zebrafish NTT was custom synthesized by WuXi AppTec (purity ≥95%; Shanghai, China). PNU282987 (CAS no. 711085-63-1) and buspirone hydrochloride (CAS No. 33386-08-2) were purchased from Tocris Bioscience (Bio-Techne®, Minneapolis, MN, United States). AZD1446 (CAS no. 1025007-04-8) was purchased from Key Organics Limited (Cornwall, UK).

### 2.2 Animals

#### 2.2.1 Mice for the SmartCube® Experiment

Male C57Bl/6 mice (8-9 weeks old; Jackson Laboratories, Bar Harbor, ME, United States) were group-housed in OPTImice® ventilated cages (4 mice/cage). Mice were acclimated to the colony room for at least 1 week prior to testing and subsequently tested at approximately 9-10 weeks of age. All animals were examined, handled, and weighed prior to the initiation of the study to assure adequate health and suitability and to minimize handling stress. During the course of the study, 12/12-h light/dark cycles were maintained. The room temperature was maintained between 20 and 23°C with a relative humidity between 30 and 70%. Chow and water were provided *ad libitum* in the home cages. Mice were randomly assigned to the treatment groups. For tolerability tests, mice were single-housed in OPTImice® ventilated cages for the duration of the study. All behavioral studies were conducted by PsychoGenics Inc. (Paramus, NJ, United States), a facility accredited by the Association for Assessment and Accreditation of Laboratory Animal Care International. The procedures were approved by the Institutional Animal Care and Use Committee in accordance with the National Institute of Health Guide for the Care and Use of Laboratory Animals (protocols 195-0513, 233-0214 and 277-1113).

#### 2.2.2 Zebrafish for NTT

Wild-type zebrafish (*Danio rerio*; strain AB) were bred and housed at Biobide (San Sebastián, Gipuzkoa, Spain) in accordance with standard procedures (Zebrafish Information Network) as described previously (Alzualde et al., 2018; Quevedo et al., 2019). In brief, the fish were maintained in a 300-L aquarium with a maximum of 1,000 fish per tank. System water was maintained at 28.5°C, pH 7–7.8, conductivity at 500–800 μS, and 80–100% oxygen and continuously filtered. The system water condition was monitored daily and regulated, if required. The fish were kept under a 14-/10-h light/dark cycle (light on at 7:30 a.m.). Adults were fed ground dry pellets (Gemma Micro 300; Sketting Zebrafish, Westbrook, ME, United States) and live food (Artemia; Catvis B.V, ‘s-Hertogenbosch, Netherlands) once a day. All behavioral experiments were performed on male and female adult zebrafish (approximately 36–52 weeks post fertilization) in accordance with European standards of animal welfare on animal use for scientific purposes (2010/63/EU), compiled with national regulations for the care of experimental animals, and were approved as described in national regulations (RD 53/2013) by local and regional committees: PRO-AE-SS-121 and PRO-AE-SS-134.

#### 2.2.3 Rats for Neurotransmitter Assay

Adult male Sprague Dawley rats (200—225 g body weight) were purchased from Charles River UK, Ltd. (Kent, United Kingdom) and were housed at the University of Birmingham animal facility, which has a procedure establishment license issued by the Secretary of State and conforms to all relevant United Kingdom legislation. The animals were terminated in accordance with schedule one procedures issued by the UK home office.

### 2.3 Tolerability

Tolerability tests were conducted by both manually scored observations and open-field activity tests to ensure that the doses used for the SmartCube^®^ test did not have any adverse effects on basic physiology and behavior. In brief, mice were single-housed prior to the test and evaluated for baseline body weight, body temperature, and other parameters. Animals exhibiting abnormal parameters were removed from the tolerability test, and the remaining mice were randomly assigned to the treatment groups, balanced by their body weight and body temperature. On day 1, the mice were i. p. injected with saline (vehicle) or a test compound at 10 ml/kg body weight. Then, body temperature was measured at 15 min, 4 h, and 24 h and body weight on days 1 and 2. Neurological and motor parameters were evaluated at 15 min, 2 h, and 4 h after administration. The list of parameters is provided in Supplementary Figure S1 in Supplementary Material S1. Behaviors that were significantly different from the vehicle-treated mice were considered abnormal.

In addition, within 5 min after the 15-min observation period, mice were placed in open-field chambers for 30 min to determine their general motor activity (distance traveled), ambulatory time, and number of rears. The open-field chambers were made of Plexiglas (27.3 × 27.3 × 20.3 cm; Med Associates Inc, St Albans, VT, United States) surrounded by infrared photobeam sources (16 × 16 × 16 beams). Horizontal activity (distance and time traveled) and vertical activity (number and frequency of rears) were measured by consecutive beam breaks. At the end of each open-field test session, the chambers were thoroughly cleaned with NOLVASAN® solution (Zoetis Services LLC, Parsippany, NJ, United States). Four mice were used per treatment condition. Mice were terminated after completion of the last tolerability observation.

All compounds were diluted in saline, and the pH was adjusted to approximately 7.0 with HCl or NaOH on the day of the experiment. The doses of the chemicals tested were as follows: nicotine (0.25, 0.5, and 1 mg/kg body weight), cotinine (2.5, 5, 10 mg/kg body weight), anatabine (1, 2, and 4 mg/kg body weight), AZD1446 (0.1, 0.3, 1, 3, and 10 mg/kg body weight), PNU282987 (0.1, 1, and 10 mg/kg body weight). The doses were calculated based on the free base molecular weight of the compounds.

### 2.4 SmartCube^®^ Behavioral Profiling

The SmartCube^**®**^ system is a unique mouse behavior profiling system developed by PsychoGenics Inc. It extracts over 2000 spontaneous and challenge-induced behavioral features during a session (Alexandrov et al., 2015; Alexandrov et al., 2016). The recorded behavioral parameters are then compared against the behavioral profiles of marketed reference compounds in the database, and the test compounds are classified into known drug classes using PsychoGenics’ proprietary bioinformatics algorithms. In brief, mice were i. p. injected with vehicle or test compound at 10 ml/kg body weight and placed in the SmartCube^**®**^ arena (24 cm × 25 cm) 15 min later. Spontaneous and stimulus-induced behaviors of mice were recorded using force sensors distributed throughout the arena during a 45-min test session. In addition, three high-resolution video cameras provided a constant 3-dimensional (3D) view of the mouse behavior in the SmartCube^**®**^ arena throughout the testing period. The bedding was vacuumed, and the arena was cleaned with NOLVASAN^**®**^ solution between each run. Data from the SmartCube^**®**^ test were processed using PsychoGenics’ proprietary Computer Vision feature extraction, Bayesian probabilistic density models, and data mining algorithms, trained on a large library of reference compounds with known therapeutic indications to predict the underlying class of each test compound (Alexandrov et al., 2015; Alexandrov et al., 2016). Twelve mice were tested per condition. Mice were terminated after the completion of the SmartCube^**®**^ test.

The doses of the tested chemicals were chosen based on the tolerability test findings as follows: nicotine (0.125, 0.25, and 0.5 mg/kg body weight), cotinine (0.25, 0.5, 1, 2.5, 5, and 10 mg/kg body weight), anatabine (0.5, 1, and 2 mg/kg body weight), AZD1446 (0.01, 0.03, 0.1, 0.3, 1, 3, and 10 mg/kg body weight), PNU282987 (0.1, 0.3, 1, 3, and 10 mg/kg body weight). The doses were calculated based on the free base molecular weight. The doses were selected based on the tolerability test results.

### 2.5 Zebrafish NTT

Adult male and female wild-type zebrafish were treated with the compounds for 20 min in a final volume of 50 ml in a 250-ml treatment beaker one fish at a time. The fish were briefly rinsed in fresh system water, and then immediately transferred to a trapezoidal tank (14.6 cm height x 5.5 cm width x 27.9 cm top length and x 23.6 cm bottom length) filled with 1.5 L system water. The behavior of the fish was monitored for the next 5 min by using the Noldus EthoVision XT system (Wageningen, Netherlands), with the camera placed approximately 1 m from the test tank. The part of the tank filled with water (11.5 cm height) was virtually divided into top, center, and bottom of equal heights (approximately 3.8 cm per segment) for the analysis. The average time spent at the top and bottom portions of the tank was analyzed to determine the anxiety-like behavior of fish. The average total distance travelled and freezing time were calculated to determine the effects of the compounds on the general behavior of fish. Freezing was defined by a complete cessation of movement except for gills and eyes (Kalueff et al., 2013). A minimum of 12 fish (6 females and 6 males) per condition were used for the study. The experimenter was blind to the test conditions. Any fish that stayed immobile for longer than 200 s out of a total of 5-min test period were considered as an outlier as it was generally >2 standard deviations away from the mean and excluded from the analysis. Three fish from vehicle control, one fish from 10 mg/L anatabine, and three fish from 100 mg/L buspirone were removed from the final analysis, but these changes did not alter the significance of statistical results.

The test concentrations were determined by first testing the compounds at 30 mg/L. If the fish tolerated the dose (as determined by the lack of abnormal behavior such as tail or body tremors or floating at the surface of the water), then higher doses were tested. If not, the dose was reduced until no obvious signs of tolerability problems were observed. The test concentrations for the NTT were as follows: nicotine (0.3, 1, 3, and 10 mg/L; equivalent to 2, 6, 19, and 62 µM), cotinine (30, 100, and 300 mg/L; equivalent to 171, 568, and 1705 µM), anatabine (0.3, 1, 3, and 10 mg/L, equivalent to 2, 6, 19, and 63 µM), and buspirone (10, 30, and 100 mg/L; equivalent to 26, 78, and 259 µM). The concentrations were calculated based on the free base molecular weight. Buspirone—a clinical anxiolytic drug used acutely and chronically to investigate the change in the anxiety-like behavior (Maximino, et al., 2011; Maximino, et al., 2013)—was included as a positive control.

### 2.6 *In vitro* Neurotransmitter Release Assay

*In vitro* neurotransmitter assays using crude synaptosome preparations were conducted by Gifford Biosciences Limited (Birmingham, UK) based on the previously described protocols (Clarke and Reuben, 1996; Gifford et al., 2000). In brief, male Sprague Dawley rats (200–225 g) were terminated by cervical dislocation followed by decapitation. The striatums or hippocampi were dissected and homogenized in ice-cold 0.32 M sucrose using a Dounce homogenizer. The homogenates were centrifuged at 100 x g for 5 min to pellet cell debris. The supernatants were collected and centrifuged at 17,000 x g for 10 min at 4°C to pellet crude synaptosomes. The pellets were resuspended in 5 ml Krebs buffer, pH 7.4 (in mM (pH 7.4): 120 NaCl, 3.3 KCl, 1.2 MgSO_4_, 1.3 CaCl_2_, 1.2 K_2_HPO_4_, 25 HEPES, 11 glucose, 0.01 ascorbic acid, 0.025 pargyline, and 0.1% BSA containing 2 μCi/ml [^3^H]norepinephrine for hippocampal synaptosomes or 1 μCi/ml [^3^H]dopamine for striatal synaptosomes and incubated for 15 min at 35°C with gentle shaking.

The [^3^H]norepinephrine- or [^3^H]dopamine-treated crude synaptosomes were loaded onto closed filter chambers containing Whatman® Grade GF/C Glass Microfiber filters (Sigma-Aldrich, Merck KGaA, Darmstadt, Germany) and placed in a superfusion system. Preoxygenated Krebs buffer was perfused through the chambers at a rate of 1 ml/min at 37 °C using an 8-channel peristaltic pump. To ensure an even flow over the synaptosomal bed, trapped air bubbles were removed from the filters prior to collection of the fractions. After a superfusion period of 40 min, three basal fractions (1.5 ml/fraction) were collected first, followed by three fractions (1.5 ml/fraction) containing the test compound. Two additional fractions were collected in the presence of 30 mM KCl to depolarize the synaptosomes. A 0.4-ml aliquot of each fraction was then transferred to a counting plate, and a scintillation cocktail was added to measure the radioactivity using a Wallac® TriLux 1450 MicroBeta counter (PerkinElmer Life Sciences, Zaventem, Belgium). Once all fractions were collected, the filters holding the crude synaptosome samples were removed and dried overnight at room temperature. On the following day, the scintillation cocktail was added and the filters were counted to determine residual radioactivity.

Compound-evoked release of neurotransmitters was calculated by subtracting the counts per minute (CPM) in the two basal fractions collected immediately prior to compound addition from those in the two fractions collected immediately following compound addition. The compound-evoked release was then expressed as a percentage of the basal release from that chamber. Potassium-evoked release was calculated by subtracting the CPM in the fraction immediately prior to KCl addition from the CPM in the three fractions immediately following potassium addition. Stimulated release was calculated as the percentage of basal release for that chamber. The increase in stimulated release above the baseline (no compound) release for that experimental run was determined, and the latter values were plotted on the graphs. Dose–response curves were determined using non-linear curve fitting in Prism (GraphPad Software, Inc, San Diego, CA, United States). All experiments were repeated at least three times.

### 2.7 *In vitro* nAChR Functional Assay

Electrophysiological responses were recorded using an automated patch-clamp Patchliner Octo® system (Nanion Technologies, Munich, Germany) equipped with two EPC-10 Quadro patch-clamp amplifiers (HEKA Elektronik, Lambrecht, Germany) as described by (Alijevic et al., 2020). In brief, Chinese hamster ovary (CHO) or human embryonic kidney-293 (HEK-293) cells stably expressing human nAChRs (Charles River Laboratories, Wilmington, MA, United States) were maintained in DMEM/F12 medium (Gibco, Thermo Fisher Scientific, Waltham, MA, United States) supplemented with 10% heat-inactivated fetal bovine serum (FBS; Gibco) and penicillin–streptomycin (100 U/mL and 0.1 mg/ml, respectively; Sigma-Aldrich, St. Louis, MO, United States) at 37°C in 5% CO_2_ and 70% humidity. The following selection antibiotics were used for the cell lines: G418 (0.25 mg/ml; Sigma-Aldrich) and Zeocin^TM^ (0.4 mg/ml; InvivoGen, San Diego, CA, United States) for α7/Ric3 nAChR and α3β4 nAChR cells; puromycin (8 μg/ml; InvivoGen) and hygromycin B (0.4 mg/ml; Gibco) for α4β2 nAChR cells; and G418 (0.5 mg/ml), puromycin (0.25 μg/ml), and hygromycin B (0.02 mg/ml) for α6/3β2/β3 nAChR cells. The cells were used for characterizing nAChR pharmacology, because no endogenous ionotropic nicotinic receptors are found (Roncarati et al., 2008; Papke and Smith-Maxwell, 2009; Kirsch et al., 2016; Scheffel et al., 2018). The subunit distributions in the cells have been previously described (Alijevic et al., 2020). The human nAChRs were selected due to the lack of commercially available nAChR expression systems for mouse or zebrafish. The translatability of findings between zebrafish and human nAChR activities have been previously reported (Papke and Smith-Maxwell, 2009; Papke et al., 2012; Alijevic et al., 2020).

On the day of the experiment, nAChR-expressing cells were suspended in extracellular solution (in mM: 140 NaCl, 4KCl, one MgCl_2_, two CaCl_2_, five glucose, and 10 HEPES, adjusted to pH 7.4 with NaOH (298 mOsmol)), then placed in the Patchliner Octo® system. The internal solution contained in mM: 50 KCl, 60 kF, 10 NaCl, 20 EGTA, and 10 HEPES, adjusted to pH 7.2 with KOH (285 mOsmol). A seal-enhancer solution (in mM: 80 NaCl, 3 KCl, 10 MgCl_2_, 35 CaCl_2_, and 10 HEPES (pH 7.4, adjusted with HCl; 298 mOsmol) was used and replaced with the external solution once the whole-cell configuration was established. For activating the nAChRs, cells were stimulated with 5–10 μL of the test compounds in 0.3% DMSO, applied at 114 μL/s, followed by a washout using 120 μL external solution. Their response was recorded at a holding potential of−70 mV and a sampling rate of 20 kHz and filtered at 3 kHz using the PatchControlHT software (Nanion Technologies, v2.01.31) in combination with the Patchmaster software (HEKA Elektronik, v2×90.4 beta). Data were analyzed using the Patchmaster software and corrected for leak current. The data acceptance criteria were as follows: seal resistance >100 MΩ; seal resistance loss variation <50%; access resistance <20 MΩ; and minimum current amplitude elicited by maximal effect concentration acetylcholine >50 pA. Offline data analysis was performed in OpenOffice™ (v4.1.2; The Apache Software Foundation, Wakefield, MA, United States). Data are presented as mean ± S.D. All experiments were performed at room temperature (24°C) and repeated at least three times. Igor Pro (v6.2.2.2; WaveMetrics, Lake Oswego, OR, United States) or Prism (v8.2.1) were used for assessing the concentration–response curves.

### 2.8 *In vitro* Molecular Target Profiling

One hundred sixty five molecular targets were selected based on various references and databases. Majority of targets were selected by using SuperPred database as a guide for known and predicted targets of the three compounds (Nickel et al., 2014). SuperPred is a publicly accessible database that provides both experimentally reported drug–target interactions (DTIs) and predicted DTIs derived by a molecular similarity approach, covering a total of 665,000 DTIs connecting 31,000 compounds and 1800 targets (Nickel et al., 2014). This database was chosen because of its comprehensive coverage for nicotine, anatabine, and cotinine compared to other databases (Fang et al., 2017). Additional targets were included based on previous in-house proteomics and SmartCube^**®**^ investigations, the abuse potential guidelines published by the United States Food and Drug Administration in 2017 (U.S. Department of Health and Human Services Food and Drug Administration (FDA) Center for Drug Evaluation and Research (CDER), 2017), and preclinical drug safety screening guidelines (Whitebread et al., 2005; Bowes et al., 2012). Combining the results of these resources, nicotine, cotinine, and anatabine were tested in technical duplicates against 175 assays including, for example, 86 GPCRs, 23 ion channels, 7 transporters, 15 kinases, and 35 other enzymes.

All binding and functional assays for molecular target characterization were conducted by Eurofins Cerep SA (Celle-Lévescault, France) and Eurofins Panlabs Discovery Services Taiwan, Ltd. (New Taipei City, Taiwan) using their standard *in vitro* binding and functional assays (Supplementary Material S2). A single concentration of each compound (10 µM in 0.1% DMSO) was used for the initial screen, followed by a full dose–response analysis for those targets for which the compounds showed an effect greater than 50%. Negative values were considered to be an artifact arising from, for example, compounds interfering with the assay readout. The initial dose was selected based on our findings that 0.5 mg/kg nicotine showed an effect in the SmartCube^**®**^, which corresponded to a plasma concentration of approximately 5–6 μM according to the pharmacokinetics data reported by Petersen et al. (1984).

The radioligand displacement binding assays employed the gold standard filtration method using membrane preparations from stable cell lines (HEK-293 or CHO cells) expressing human or rodent target proteins to determine the interaction of the compounds with specific receptors, channels, and transporters. For this purpose, the competitive binding of test compounds against a [^125^I]- [^3^H]-, or [^35^S]-labelled agonist and/or antagonist was determined. The specific list of radiolabeled ligands and experimental conditions are summarized in Supplementary Material S2.

### 2.9 Statistical Analysis

For the tolerability tests, passive signs and manipulation responses were analyzed for effects of the treatment by the Kruskal–Wallis test. Body temperature and body weight data were analyzed by two-way repeated measures analysis of variance (ANOVA). The total distance and time traveled and number and frequency of rears in the open-field activity test were analyzed by one-way ANOVA, followed by the Tukey *post-hoc* test. For the analyses of the zebrafish NTT data, one-way ANOVA was used, followed by Dunnett’s multiple comparison test, if it passed the Shapiro-Wilk normality test. If the data set did not pass the normality test, then Kruskal–Wallis test followed by Dunn’s multiple comparison test was used. An effect was considered significant if *p* < 0.05. The half maximal effective concentration (EC_50_) and half-maximal inhibitory concentration (IC_50_) for the receptor pharmacology and neurotransmitter release assay were determined by using nonlinear regression analysis. Statistical analyses were conducted using GraphPad Prism.

## 3 Results

### 3.1 SmartCube^®^ Behavioral Profiling and Classification

To investigate a wide range of neurobehavioral effects, nicotine, cotinine, and anatabine were tested in the SmartCube^**®**^ system after a single i.p. injection in mice. Two reference compounds, AZD1446 and PNU282987, were included as α4β2 and α7 nAChR-specific agonists, respectively. The treatment protocol was chosen to be consistent with the treatment protocol used to establish the SmartCube^**®**^ reference database. To determine the test doses, three doses of each compound were tested for tolerability. The results indicated that the highest dose (10 mg/kg) of cotinine, AZD1446, and PNU282987 were well tolerated and thus, 10 mg/kg was selected as the highest dose to be tested on the SmartCube^**®**^ system (Supplementary Material S3; Supplementary Figure S2 in Supplementary Material S1). For anatabine and nicotine, the highest dose tested (4 mg/kg and 1 mg/kg, respectively) decreased the body temperature of the mice 15 min after the injection (Supplementary Figure S3 in Supplementary Material S1; main treatment effect: F (6, 21) = 12.619; *p* < 0.001; treatment × time interaction effect: F (6, 21) = 11.755; *p* < 0.001; LSD post hoc: *p* < 0.05 for both). Thus, a lower dose (2 mg/kg and 0.5 mg/kg, respectively) was chosen to be tested as the highest dose for the SmartCube^**®**^ experiment.

**FIGURE 1:**
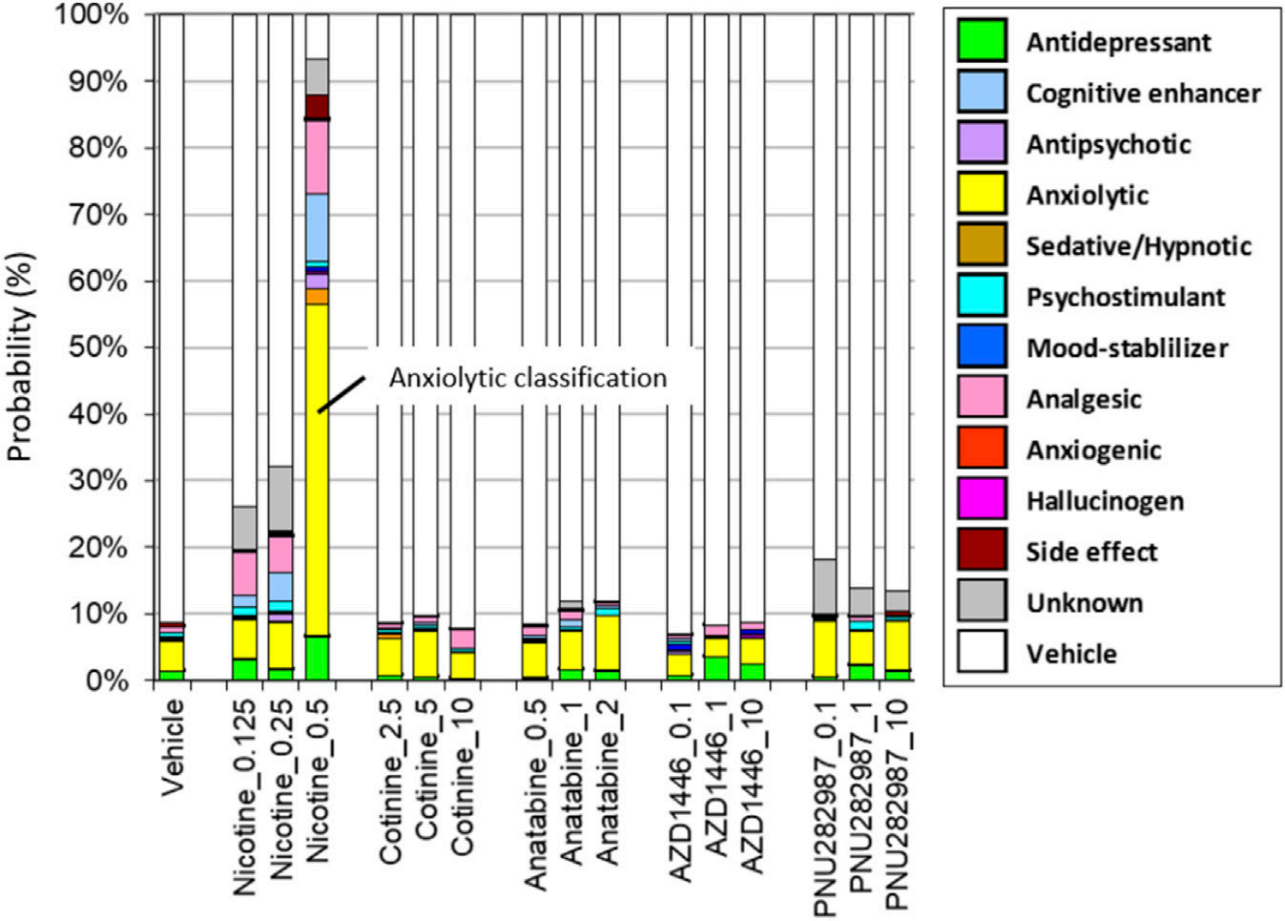
Drug classifications of the plant alkaloids. The drug classifications of nicotine, cotinine, anatabine, and nAChR reference compounds (AZD1446 and PNU282987) determined by using the SmartCube^**®**^ system in mice are presented. Only nicotine induced anxiolytic-like behavioral signature (yellow bar). Neither the free base nor citrate form of anatabine showed any changes in behavior. Thus, only the free base data are shown for anatabine. The doses are indicated on the *x*-axis as mg/kg. The color code is described in the figure legend on the left. N = 12 mice.

Among the compounds tested on the SmartCube^**®**^ system, only nicotine at the highest dose (0.5 mg/kg body weight) showed anxiolytic-like behavior features in mice. Other compounds did not induce any behavioral changes that significantly differed from those induced by the vehicle control (Figure 1).

### 3.2 Effects of Alkaloids on Zebrafish NTT Response

**FIGURE 2:**
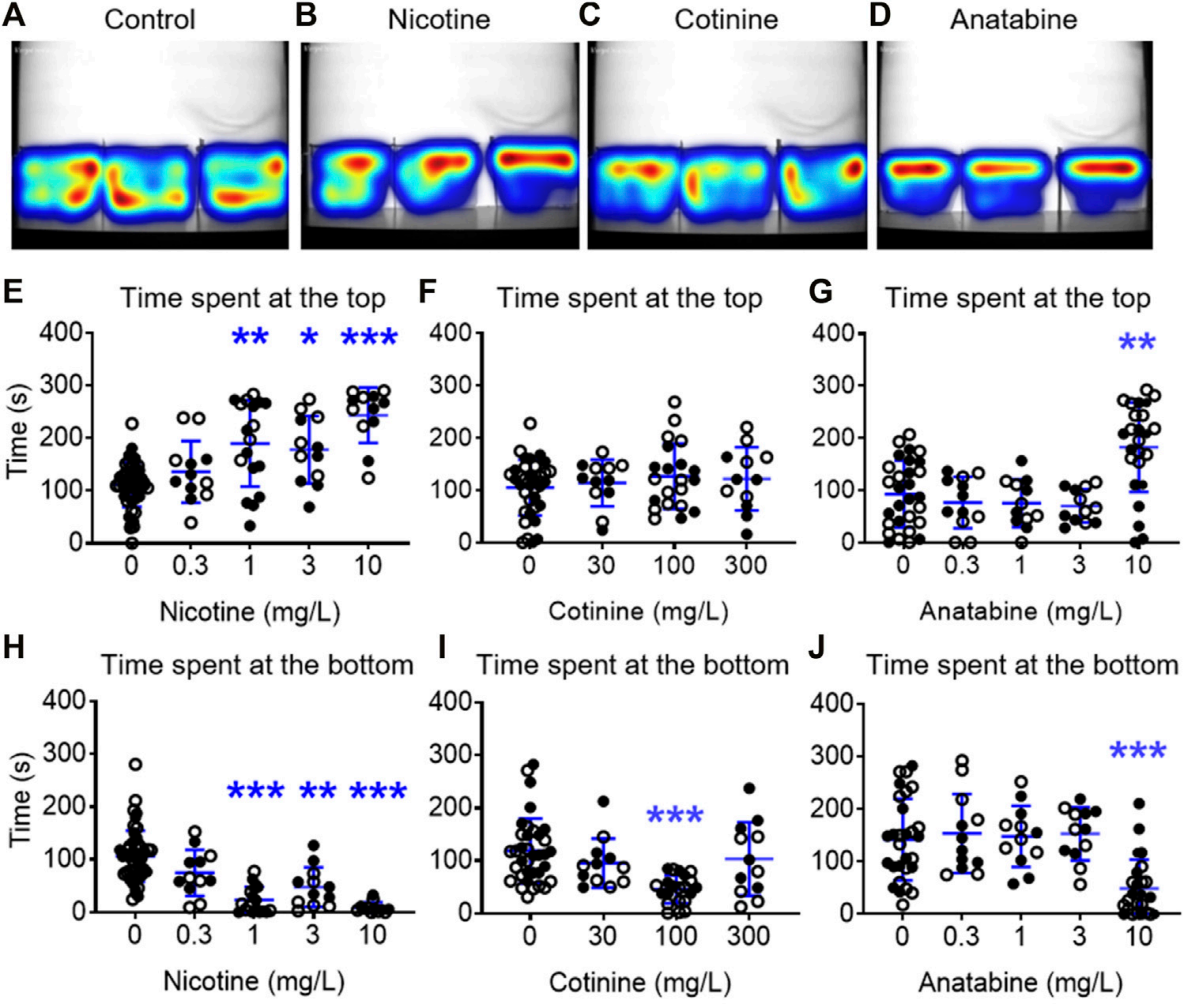
Effects of alkaloids on anxiety-like behavior in zebrafish. Heatmaps of the general activity of zebrafish after **(A)** vehicle, **(B)** nicotine (1 mg/L), **(C)** cotinine (100 mg/L), or **(D)** anatabine (10 mg/L) treatment are shown. Nicotine increased the time spent at the top and decreased the time spent at the bottom for the three highest doses, 1, 3, and 10 mg/L **(E, H)**. Cotinine decreased the time spent at the bottom only at 100 mg/L and did not affect the time spent at the top **(F, I)**. Anatabine increased the time spent at the top and decreased the time spent at the bottom at only the highest dose tested (10 mg/L; **(G, J)**. Each individual circle represent one zebrafish. Solid circles = males; open circles = females; *n* = 12–36; *p < 0.05 and ***p < 0.001. Data are presented as mean ± S.D.

The zebrafish NTT was used to assess the anxiolytic-like effects of three alkaloids. In this experiment, zebrafish were placed in a beaker containing nicotine, cotinine, or anatabine for 20 min, then placed in a novel tank. The top three concentrations of nicotine (1, 3, and 10 mg/L) increased the time spent at the top and reduced the time spent at the bottom (Figures 2B, E, H; H (4) = 36.38; *p* < 0.001; Dunn’s post hoc: *p* = 0.001, 0.013, <0.0001 for 1, 3, and 10 mg/L, respectively for the time spent at the top; H (4) = 59.66; Dunn’s post hoc: *p* < 0.0001 for 1 and 10 mg/L, *p* < 0.004 for 3 mg/L for the time spent at the bottom). Zebrafish exposed to 100 mg/kg cotinine spent less time at the bottom of the tank, but other concentrations had no effect (Figures 2C,F,I **;** H (3) = 23.86; *p* < 0.0001; Dunn’s post hoc: *p* < 0.0001). The time spent at the top was not affected by any doses of cotinine. Anatabine increased the time spent at the top and reduced the time spent at the bottom only at the highest dose tested (Figures 2D,G,J; H (4) = 24.40; *p* < 0.0001; Dunn’s post hoc: *p* = 0.001 for the time spent at the top; H (4) = 30.73; *p* < 0.0001; Dunn’s post hoc: *p* < 0.0001 for the time spent at the bottom). The anxiolytic reference compound buspirone increased the time spent at the top for all three doses tested and reduced the time spent at the bottom for the middle two doses (Supplementary Figures S4A, B in Supplementary Material S1; F (3, 58) = 22.60; *p* < 0.0001; Dunnett’s post hoc: *p* = 0.0002, <0.0001, 0.0078 for 10, 30, and 100 mg/L, respectively for the time spent at the top; H (3) = 33.21; *p* < 0.0001; Dunn’s post hoc: *p* = 0.014 and <0.0001 for 10 and 30 mg/L, respectively for the time spent at the bottom), supporting the validity of the zebrafish NTT to detect anxiolytic-like compounds.

**FIGURE 3:**
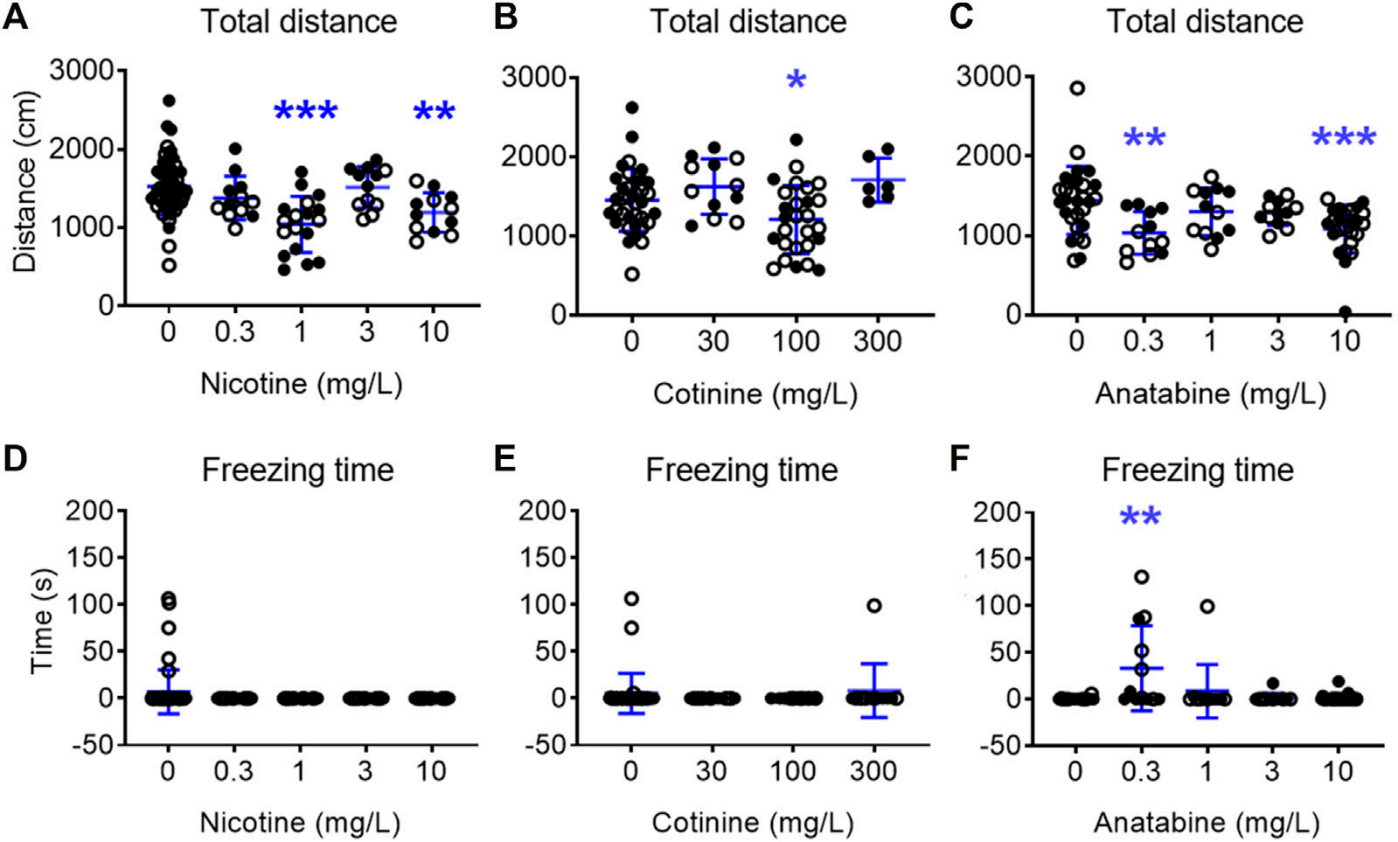
Effects of alkaloids on general movement in zebrafish. Total distance traveled over 5 min test period for **(A)** nicotine, **(B)** cotinine, and **(C)** anatabine and freezing time for **(D)** nicotine, **(E)** cotinine, and **(F)** anatabine are presented. A slight reduction in the movement was detected for fish exposed to nicotine at 1 and 10 mg/L, cotinine at 100 mg/L, and anatabine at 0.3 and 10 mg/L. Freezing time was only increased by 0.3 mg/L anatabine treatment. Each individual circle represent one zebrafish. Solid circles = males; open circles = females; *n* = 12–36; *p < 0.05 and ***p < Data are presented as mean ± S.D.

When the general behavior was examined, the fish treated with 1 and 10 mg/L nicotine showed decreased total distance traveled, but showed no freezing response (Figures 3A,D in Supplementary Material S1; F (4, 100) = 8.520; *p* < 0.0001; Dunnetts’ post hoc: *p* < 0.0001 for 1 mg/L, *p* = 0.010 for 10 mg/L for total distance traveled). Total distance traveled in fish exposed to 100 mg/L cotinine was reduced without affecting their freezing time (Figures 3B,E in Supplementary Material S1; F (3, 77) = 4.832; *p* = 0.0039; Dunnett’s post hoc: *p* = 0.049). Total distance travelled was reduced in zebrafish exposed to 0.3 and 10 mg/L anatabine (Figure 3C in Supplementary Material S1; H (4) = 19.19; *p* < 0.001; Dunn’s post hoc: *p* = 0.002 and 0.001 for 0.3 and 10 mg/L, respectively). The freezing time was increased only at 0.3 mg/L (Figure 3F; H (4) = 14.53; *p* < 0.006; Dunn’s post hoc: *p* = 0.002). The total distance traveled was decreased and the freezing time was increased at 100 mg/L buspirone (Supplementary Figures S4C, D in Supplementary Material S1; F (3, 58) = 4.785; *p* = 0.005; Dunnett’s post hoc: *p* = 0.009 for total distance; H (3) = 29.22; *p* < 0.0001; Dunnett’s post hoc: *p* < 0.0001 for freezing time), suggesting potential tolerability challenge at this very high dose of buspirone. The changes in total distance travelled for all compounds were rather small albeit significant, and the reduced activity did not always result in anxiolytic-like behavior and vice versa (e.g., 3 mg/L nicotine and 0.3 mg/L anatabine). There were also no significant differences between male and female zebrafish responses for all the parameters examined.

### 3.3 Neurotransmitter Release

**FIGURE 4:**
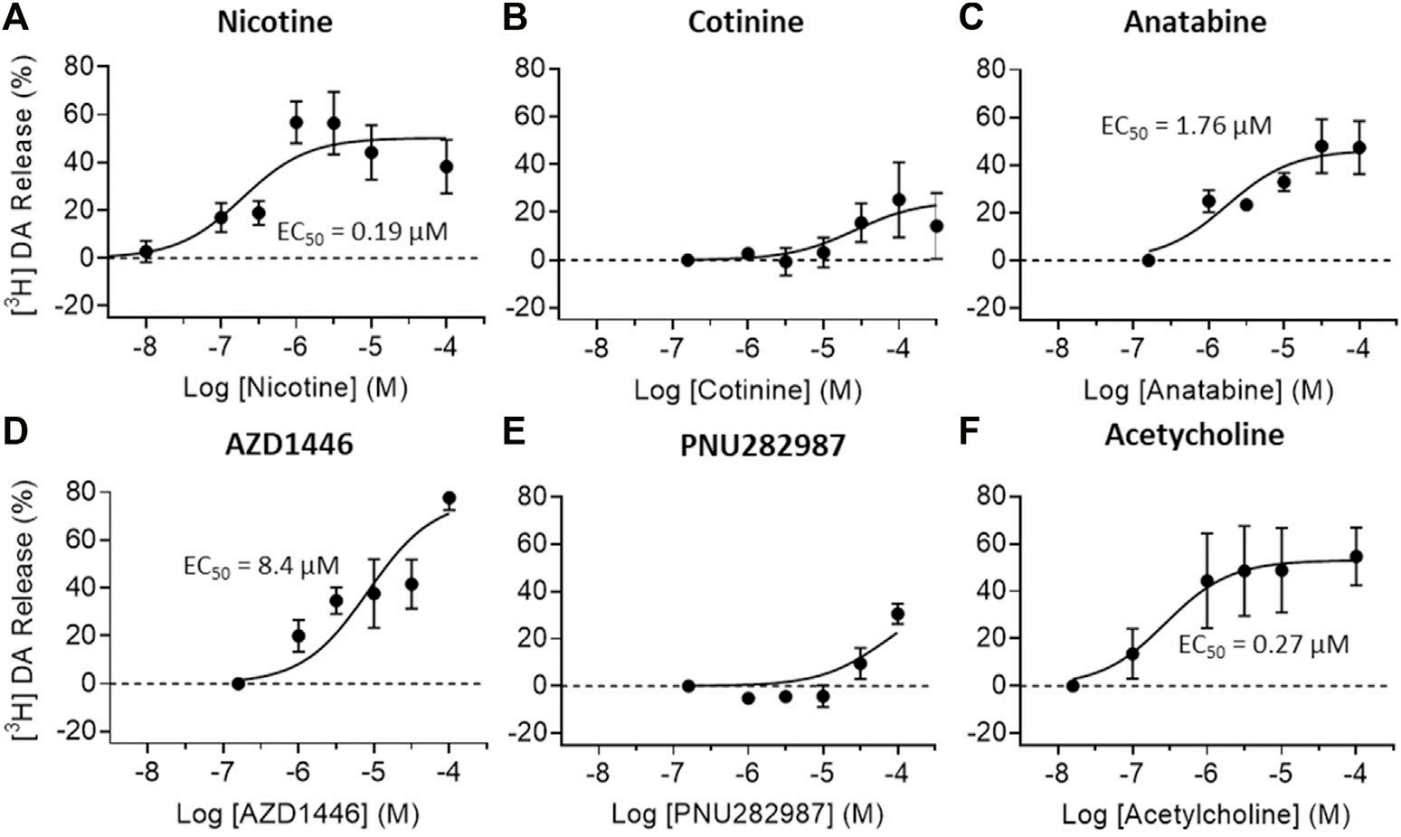
Effects of nAChR ligands on dopamine release *in vitro*. Dopamine (DA) release from crude striatal synaptosome preparations were measured after **(A)** nicotine, **(B)** cotinine, **(C)** anatabine, **(D)** AZD1446 **(E)** PNU282987, and **(F)** acetylcholine treatment. All tested compounds elicited robust DA release except for cotinine and PNU282987 at the concentrations tested. Data are presented as mean ± S.D.

**FIGURE 5:**
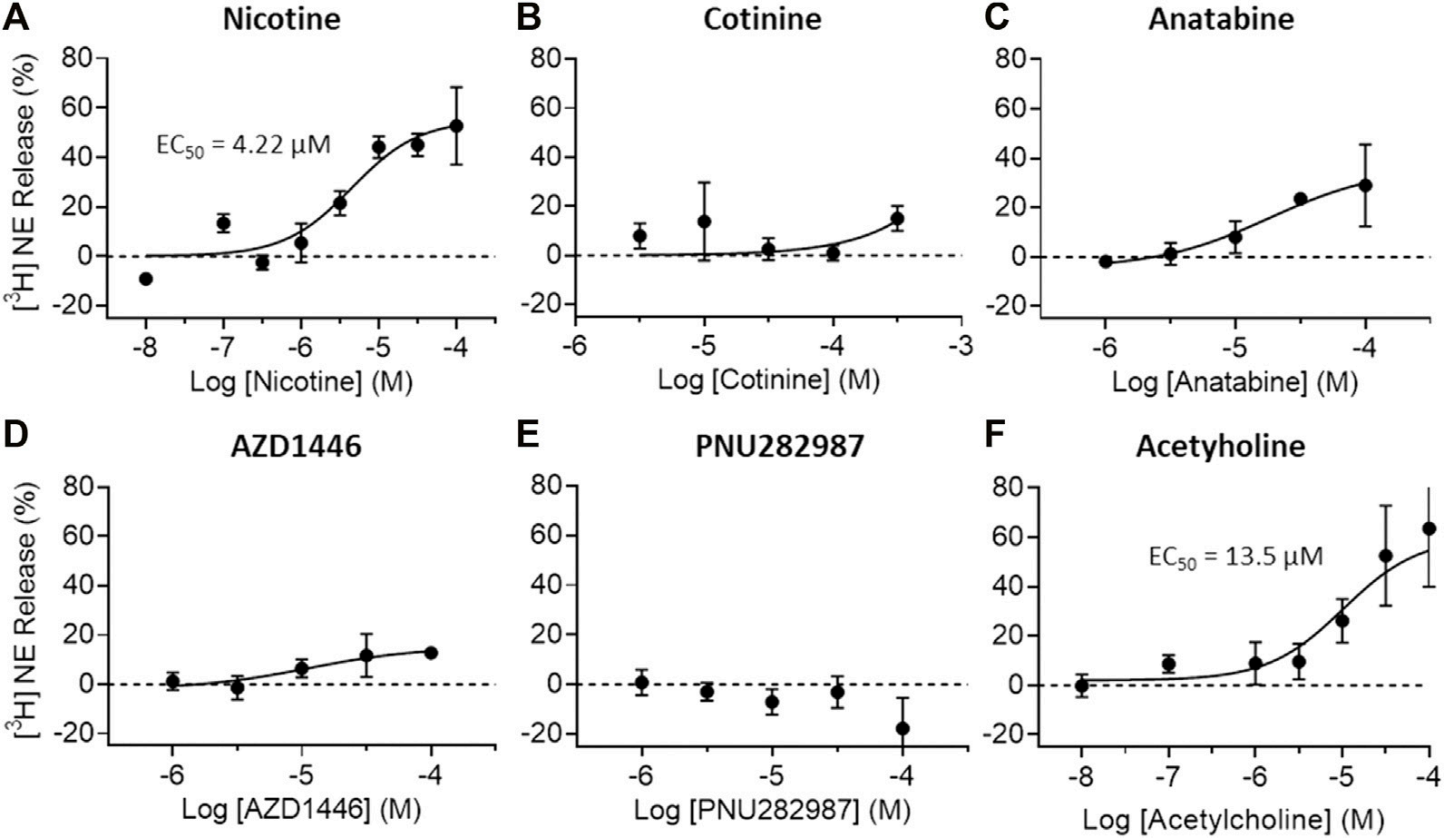
Effects of nAChR ligands on *in vitro* norepinephrine release. Norepinephrine (NE) release from crude hippocampal synaptosome preparations were measured after **(A)** nicotine **(B)** cotinine, **(C)** anatabine, **(D)** AZD1446, **(E)** PNU282987, and **(F)** acetylcholine treatment. Only nicotine and acetylcholine seem to elicit clear NE release at the concentrations tested. Data are presented as mean ± S.D.

To understand the possible regulatory role of nicotine, cotinine, and anatabine on neurotransmitter systems relevant for emotionality such as anxiety, we investigated the effects of these alkaloids on dopamine and norepinephrine release *in vitro*. The AZD1446 and PNU282987 were included as α4β2 and α7 nAChR specific reference compounds, respectively (Bodnar et al., 2005; Mazurov et al., 2012), and acetylcholine as an endogenous nAChR ligand. Our results showed that dopamine release from striatal synaptosomes was partially induced by nicotine, anatabine, AZD1446, and acetylcholine (EC_50_: 0.19, 1.76, 8.4, and 0.27 μM, respectively) (Figure 4). Cotinine and PNU282987 at higher concentrations induced 20–30% dopamine release, but the results were either too variable or not sufficiently potent to reliably assess EC_50_ values. Norepinephrine release from hippocampal synaptosomes was induced only by nicotine and acetylcholine (EC_50_: 4.22 and 13.5 μM, respectively) (Figure 5). Anatabine induced a slight increase (∼20%) in norepinephrine release at the higher doses, but its EC_50_ could not be reliably assessed due to the high variability of the data at the highest concentration tested. The α4β2 nAChR agonist AZD1446 and the α7 nAChR agonist PNU282987 induced negligible change in norepinephrine release.

### 3.4 *In vitro* Molecular Target Profiling of Nicotine, cotinine, and Anatabine

**FIGURE 6:**
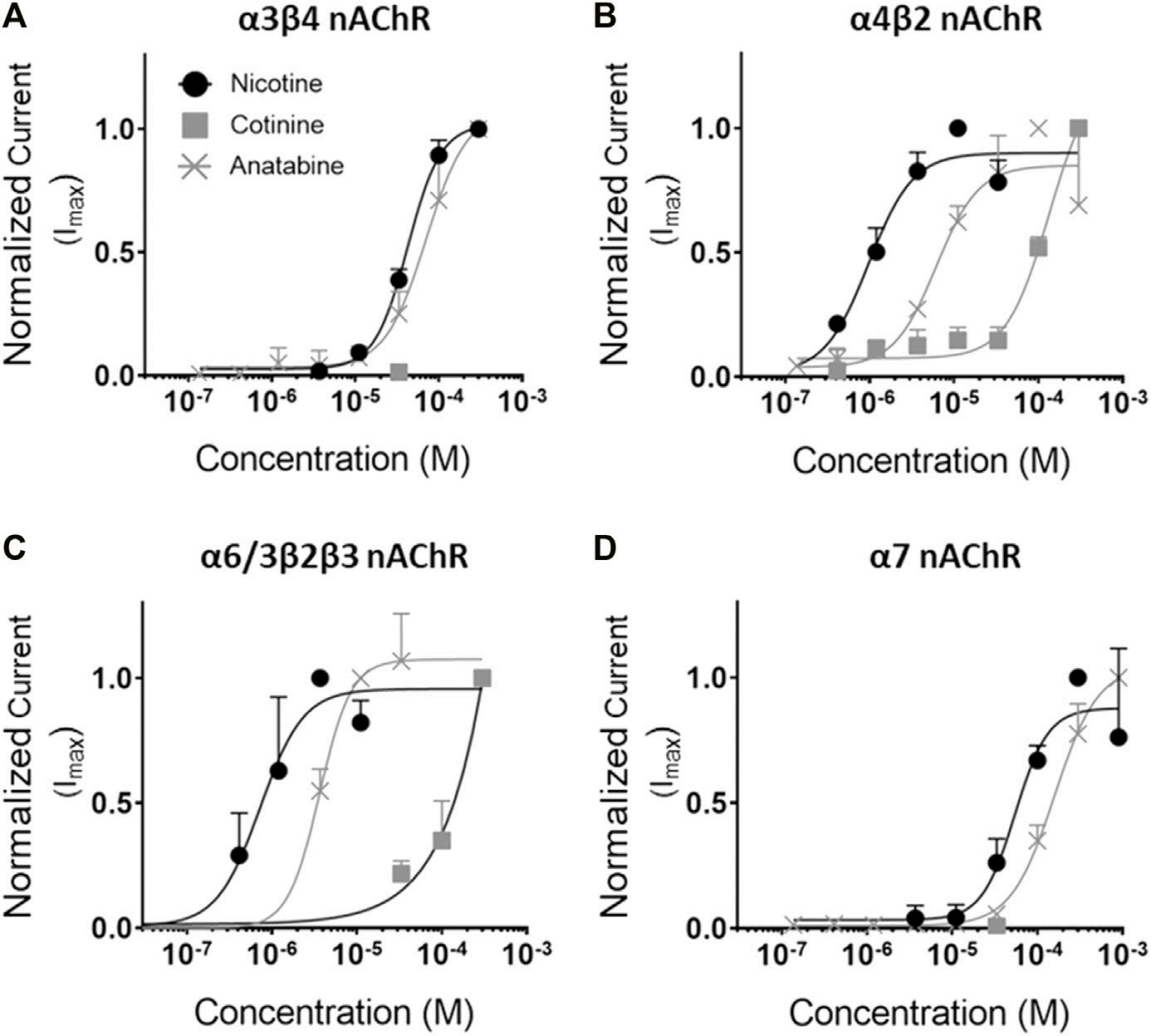
Concentration response curves of the alkaloids for various nAChRs. Functional activity of nicotine, cotinine, and anatabine were tested against **(A)** α3β4, **(B)** α4β2, **(C)** α6/3β2β3, and **(D)** α7 *in vitro*. Mean EC_50_ values (in µM) are indicated in Table 1. Nicotine = black lines with solid circles; Cotinine = grey lines with solid squares; anatabine = grey lines with crosses. Data are presented as mean ± S.D.

To understand the molecular mechanisms, dose–response binding and/or functional studies were conducted for α3β4, α4β2, α6/3β2β3, and α7 nAChR subtypes *in vitro* (Figure 6; Table 1, and Supplementary Figure S5 in Supplementary Material S1). Nicotine was a potent full agonist for α4β2 and α6/3β2β3 nAChRs (EC_50_ = 1.0 ± 0.2 and 0.7 ± 0.1 µM, respectively) and showed weak activity against α3β4 and α7 nAChRs (EC_50_ = 42.4 ± 2.2 and 54.5 ± 10.6 µM, respectively). Anatabine showed a slightly weaker potency for all receptor subtypes compared to nicotine (EC_50_ for α3β4 nAChR = 70.6 ± 8.2 µM; for α4β2 nAChR = 6.1 ± 1.4 µM; for α6/3β2β3 nAChR = 3.6 ± 0.3 µM; for α7 nAChR = 158.5 ± 11.4 µM). Cotinine was the least potent of the three alkaloids with EC_50_ for α4β2 and α6/3β2β3 nAChRs greater than 100 µM and no detectable activities for α3β4 and α7 nAChRs for the range of concentrations tested in this study. In support of this weak activity, the cotinine binding for α3β4 and α7 nAChRs were also undetectable and for α4β2 was barely detectable (Supplementary Figures S5B, E, and H in Supplementary Material S1). In contrast, nicotine and anatabine showed a strong binding affinity towards α3β4 and α4β2 nAChRs (α3β4 nAChR IC_50_ for nicotine and anatabine = 1.00 ± 0.08 and 0.96 ± 0.20 µM, respectively; α4β2 nAChR IC_50_ for nicotine and anatabine = 0.04 ± 0.002 and 0.71 ± 0.09 µM, respectively) (Supplementary Figure S5A, D in Supplementary Material S1 **,** for nicotine; 6C and F for anatabine). The EC_50_ values for α7 nAChR were barely detectable at 10 µM for both nicotine and anatabine (Supplementary Figures S5G, I in Supplementary Material S1). Binding assays could not be conducted for α6/3β2β3 nAChRs due to the lack of commercially available compounds that are specific to α6-contatining subtypes. It is worth noting that independent functional assays were conducted for each compound, and, thus, the possible roles of the compounds as non-competitive or silent agonists or allosteric modulators were not assessed.

**TABLE 1:** The EC_50_ values of alkaloids for various nAChR subtypes.

	α3β4 nAChR EC50 in µM	α4β2 nAChR EC50 in µM	α6/3β2β3 EC50 in µM	α7 nAChR EC50 in µM
Nicotine	42.4 ± 2.2	1.0 ± 0.2	0.7 ± 0.1	54.5 ± 10.6
Cotinine	No effect *	Undetermined	Undetermined	No effect*
Anatabine	70.6 ± 8.2	6.1 ± 1.4	3.6 ± 0.3	158.5 ± 11.4

The EC_50_ values of all compounds are presented as mean ± S.D. * Up to 33 μM.

**FIGURE 7:**
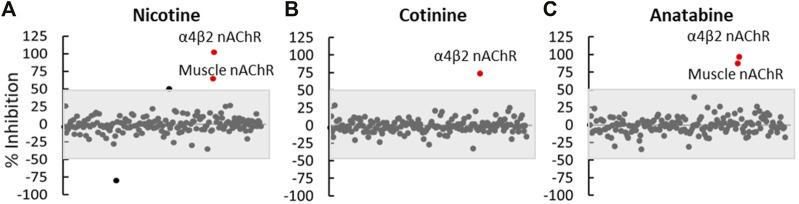
Off-target effect assessment. **(A)** Nicotine, **(B)** cotinine, and **(C)** anatabine were tested in 175 *in vitro* binding or functional assays as summarized in Supplementary Material S2. Each dot represents the outcome of one assay. The grey shaded area covers any assay that showed an effect smaller than 50%. Red dots represent assays that showed greater than 50% change by the respective compounds compared to the vehicle control. N = three repeats.

To understand potential off-target effects of these alkaloids, we selected 175 *in vitro* binding and enzymatic assays to determine the molecular target specificity of nicotine, cotinine, and anatabine based on the database and previous studies. The result indicated that all three alkaloids showed specific binding to α4β2 and muscle-type nAChR, but did not bind or regulate the activities of other molecular targets *in vitro* (Figure 7).

## 4 Discussion

In this study, we investigated the neurobehavioral effects of three alkaloids—nicotine, cotinine, and anatabine—by using two relatively high-throughput behavioral paradigms, the SmartCube® system and zebrafish NTT. We were able to demonstrate the anxiolytic-like effect of nicotine by using both systems, supporting the robustness of the finding across species. Cotinine induced a weak anxiolytic-like effect at a concentration 100-fold higher than nicotine in zebrafish, with the effect only observable when the time spent at the bottom was considered. Similarly, anatabine also induced an anxiolytic-like effect, but only at the highest tolerated concentration, which was 10-fold higher than nicotine. The relative low potency of cotinine and anatabine may have been reflected by the lack of anxiolytic-like effect detected in the SmartCube® system. The fact that cotinine did not induce a strong neurobehavioral effect suggests that the observed effect of nicotine was likely due to the direct effect of nicotine and not due to its metabolic product. Furthermore, nicotine was able to induce dopamine and norepinephrine release *in vitro*, while cotinine and anatabine mainly induced dopamine release only. Previous studies have demonstrated the translational value of dopamine and norepinephrine signaling systems among zebrafish, rodents, and humans (Singh et al., 2015; Ek et al., 2016; Feng et al., 2019). Thus, although the *in vitro* dopamine and norepinephrine release assays were conducted using rat synaptosomes, the findings should be applicable across species. The differences of these alkaloids in regulating neurobehavioral effects and neurotransmitter release may be reflected by the different levels of nAChR activation, where nicotine showed the strongest potency against almost all receptor subtypes examined. In addition, our preliminary results suggested that anatabine may not fully activate α3β4, α4β2, and α6/3β2β4 nAChRs, inducing perhaps, 40, 60, and 70% of the full receptor activity, respectively (data not shown).

Dopamine and norepinephrine are tightly regulated to control anxiety in animals (Garcia-Garcia et al., 2014; Montoya et al., 2016). It has been well-documented that activation of nAChRs induces the release of norepinephrine in the hippocampus from terminals originating in the locus coeruleus and of dopamine in the striatum from terminals originating in the substantia nigra or the ventral tegmentum (Rapier et al., 1988; Rapier et al., 1990; Sacaan et al., 1995; Clarke and Reuben, 1996). Various reports suggest that presynaptic nAChRs associated with striatal dopaminergic and hippocampal noradrenergic terminals differ pharmacologically to finely regulate their neurotransmitter release mechanisms. For example, nigrostriatal dopaminergic terminals have been suggested to have at least two types of nAChRs: *a*-conotoxin MII (α-CtxMII)-sensitive and -insensitive nAChRs (Kulak et al., 1997). The β2 subunit of nAChRs is absolutely required for both *a*-CtxMII-sensitive and -insensitive nAChR-mediated dopamine release, while the β4 and α7 subunits are not (Salminen et al., 2004). The distinguishing composition of these nAChRs is that the *a*-CtxMII-sensitive response requires the β3 and α6 subunits and is partially dependent on the α4 subunit (e.g., α6β3β2 and α4α6β3β2), whereas the *a*-CtxMII-resistant release requires the α4 subunit and is partially dependent on the α5 subunit (e.g., α4β2 and α4α5β2) (Champtiaux et al., 2002; Champtiaux et al., 2003; Luetje, 2004; Salminen et al., 2004). The contribution of α7 receptors to the control of dopamine release and, in fact, of norepinephrine release also, is mediated indirectly via an increase in glutamate release (Salminen et al., 2004; Barik and Wonnacott, 2006). Consistent with these results, Zoli et al. (2002) concluded that nicotinic binding sites expressed in rats include α4β2, α4α5β2, α6β2 (β3), and α4α6β2 (β3) nAChRs (Zoli et al., 2002). Thus, α4β2 nAChR-activating compounds, such as nicotine, anatabine, and AZD1446 used in the current study, can strongly induce dopamine release *in vitro*, while α7 nAChR agonists, such as PNU282987, induce a marginal effect.

Similarly, locus coeruleus noradrenergic neurons projecting to the hippocampus also show specific nAChR subunit compositions that can be differentially modulated by various nAChR ligands. Two populations of neurons can be distinguished on the basis of nAChR mRNA expression patterns and electrophysiological properties (Wada et al., 1989; Wada et al., 1990; Dineley-Miller and Patrick, 1992; Lena et al., 1999). One population of small cells systematically express α3 and β4 mRNAs (and often α6, β3, α5, and α4 mRNAs). Another population of cells with large soma systematically express α6 and β3 (and often α4), but not α3 and β4 mRNAs. Nicotine preferentially elicits large currents in the large cells, while cytisine preferentially elicits large currents in the small cells. This nAChR-specific and, thus, cell-type specific activation allows nicotine to more potently induce norepinephrine release than cytisine in the hippocampus, indicating that the noradrenergic terminals in the hippocampus most likely originate from the large α6-and β3-expressing cells (e.g., α6β3β2 and α4α6β3β2) in the locus coeruleus (Lena et al., 1999). This fine-tuning of norepinephrine release by receptor subtype-specific activation may explain why nicotine was uniquely classified as an anxiolytic-like compound using mice in this study, while others, such as cotinine and anatabine with no potency or low potency and partial activation (approximately 67% of nicotine; preliminary data) of α6-containing nAChRs, respectively, were not.

It is worthy to note that previous studies have reported both anxiolytic and anxiogenic effects of nicotine in other nonclinical models (Lippiello et al., 1996; Lippiello et al., 2007; Mineur et al., 2007; Terry et al., 2012; Grizzell et al., 2014; Levin et al., 2014; Grizzell and Echeverria, 2015; Terry et al., 2015; Elhassan et al., 2017; Xia et al., 2019). Similarly, nAChR antagonists, such as mecamylamine, have also been reported to possess both anxiolytic and anxiogenic properties in nonclinical studies (Zarrindast et al., 2000; Newman et al., 2001; Picciotto et al., 2002). The ability of nAChR agonists and antagonists to act as an anxiolytic or anxiogenic substance is quite complex and dependent on the regimen of administration (acute vs chronic regimens, or withdrawal), route of administration (i.p., subcutaneous, intravenous, or inhaled), and behavioral state of the experimental subjects (relaxed vs stressed) (Picciotto et al., 2002; Picciotto et al., 2015). In particular, the baseline level of endogenous acetylcholine, which can vary depending on, for example, the stress level of the animal, could be rather important to understand the drug effects (Imperato et al., 1989; Imperato et al., 1991). The changing endogenous acetylcholine levels can modify nAChR sensitization or desensitization state, which ultimately determine the drug effect on behavioral outcome (Lu et al., 1999; Giniatullin et al., 2005; Yu et al., 2014). These various factors in nonclinical models, influencing the effect of nicotine and anxiety, in general, also make it challenging to interpret their implications in human neuropharmacology and ultimately, human behavior. Thus, future research is certainly worthwhile to assess if chronic treatment of nicotinic alkaloids or other considerations can produce similar anxiolytic-like effects.

Taken together, our results indicate that nicotinic ligands can induce an anxiolytic-like effect. The differential neurobehavioral effects induced by the three alkaloids suggest a fine regulation of neurotransmitter systems orchestrated by a complex combination of various nAChR subtypes. Previous studies showing nAChR-mediated mechanisms using specific antagonists (Levin, 2002; Terry et al., 2015; Bertrand and Terry, 2018; Terry and Callahan, 2019) support these concepts outlined in this study. Although cotinine and anatabine did not induce a strong anxiolytic-like effect, cotinine, in particular was well tolerated in both fish and mice. Thus, these findings support the importance of investigating the therapeutic potential of natural compounds that are well tolerated.

## Data Availability

The raw data supporting the conclusion of this article will be made available by the authors, without undue reservation.
