# Supplementary material for: Differentiating the Neuropharmacological Properties of Nicotinic Acetylcholine Receptor-Activating Alkaloids
Source: Front Pharmacol. 2022 Mar 22;13:668065. doi: 10.3389/fphar.2022.668065 (PMC8980233; doi:10.3389/fphar.2022.668065)
Supplement: Supplementary file 1 [file Presentation1.PPTX]

## Slide 1
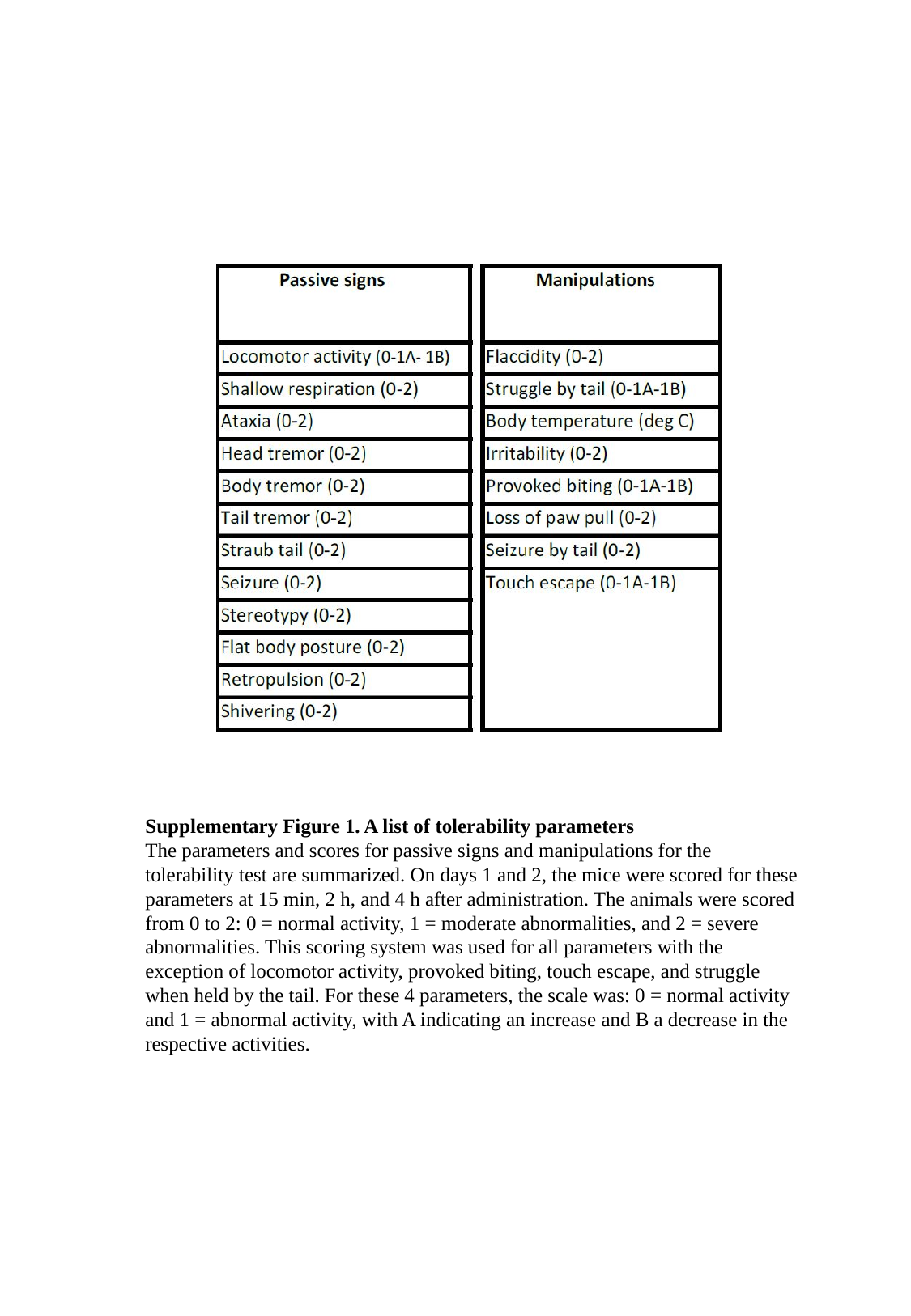

Supplementary Figure 1. A list of tolerability parameters
The parameters and scores for passive signs and manipulations for the tolerability test are summarized. On days 1 and 2, the mice were scored for these parameters at 15 min, 2 h, and 4 h after administration. The animals were scored from 0 to 2: 0 = normal activity, 1 = moderate abnormalities, and 2 = severe abnormalities. This scoring system was used for all parameters with the exception of locomotor activity, provoked biting, touch escape, and struggle when held by the tail. For these 4 parameters, the scale was: 0 = normal activity and 1 = abnormal activity, with A indicating an increase and B a decrease in the respective activities.

## Slide 2
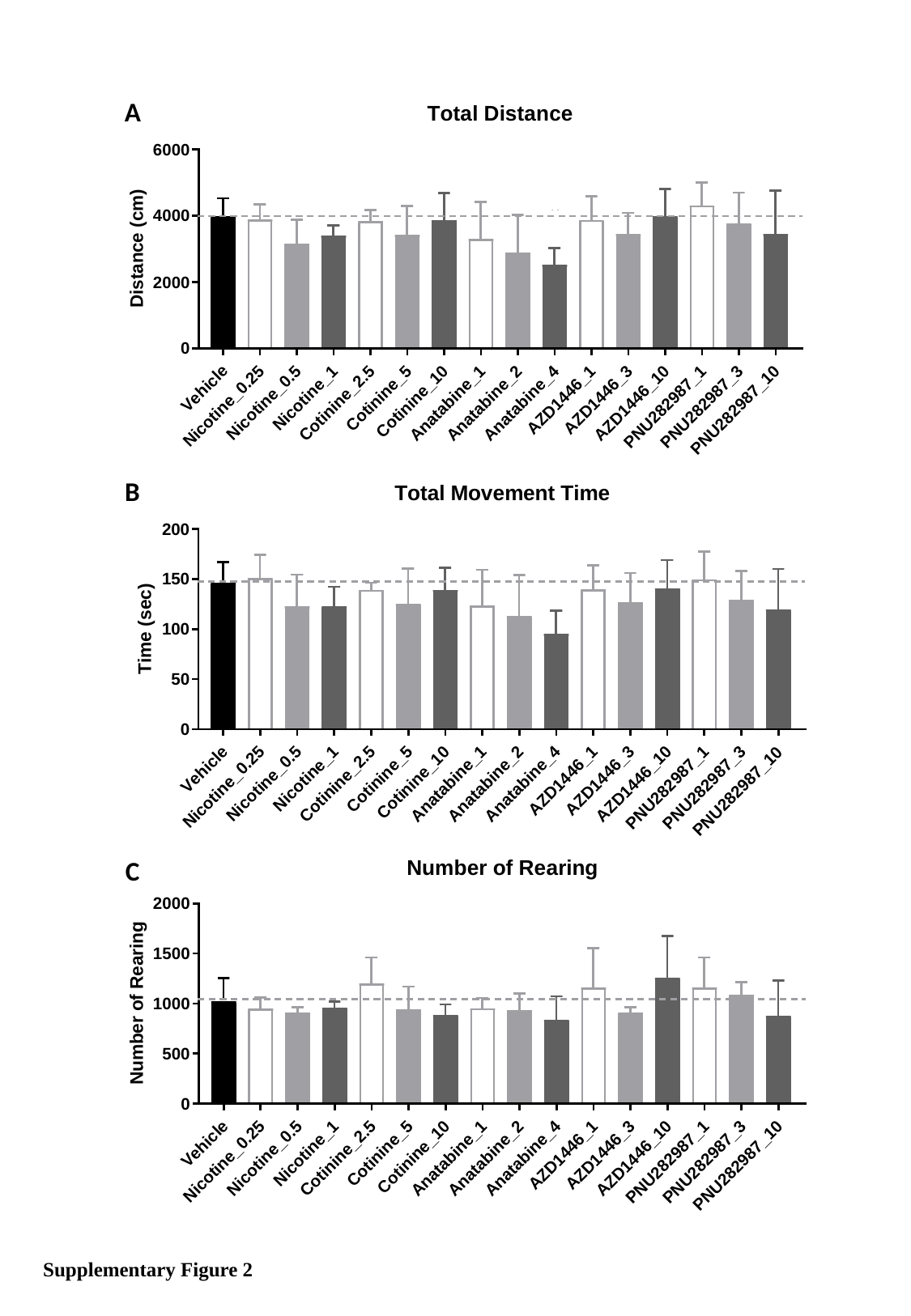

A
B
C
Supplementary Figure 2

## Slide 3
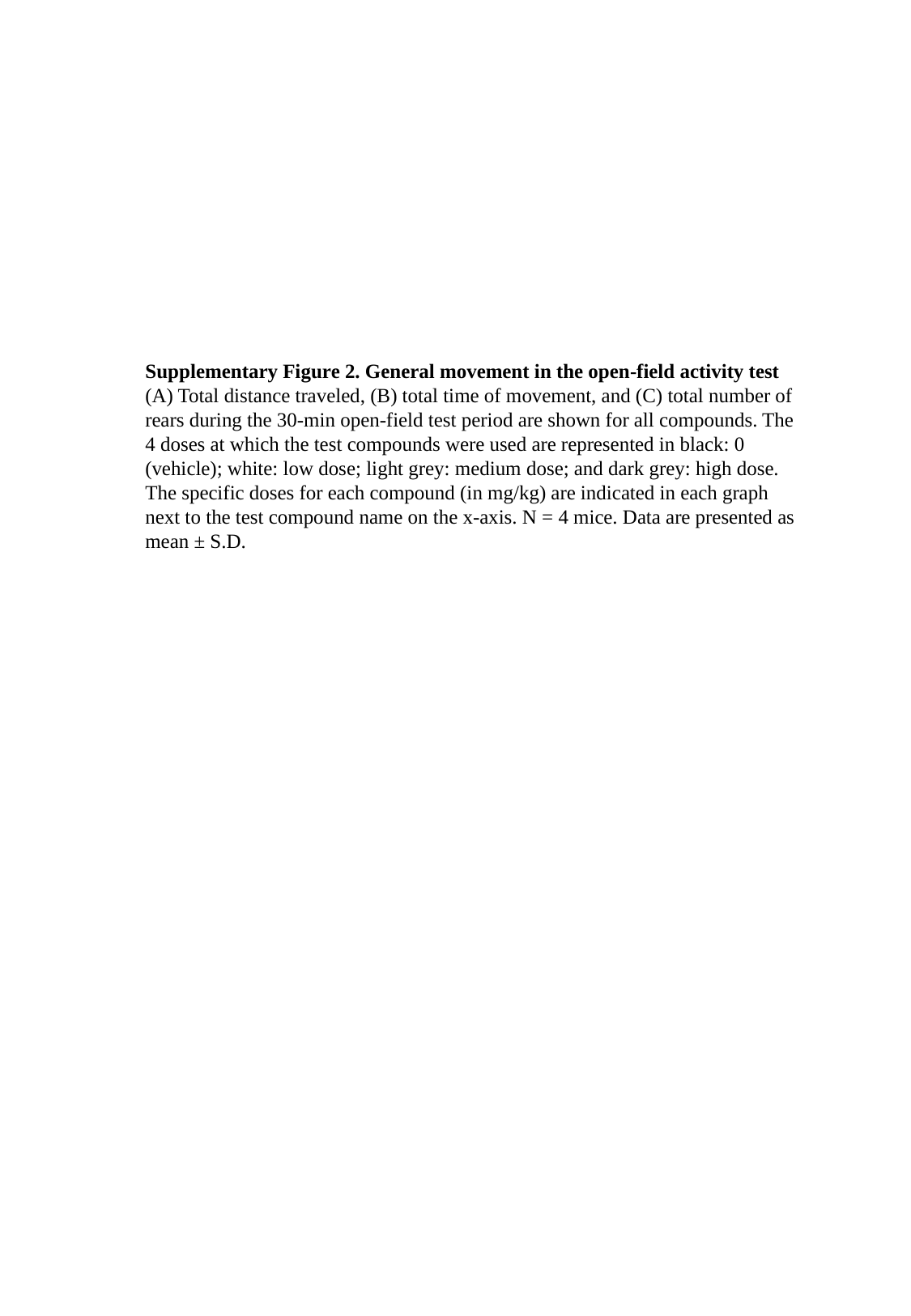

Supplementary Figure 2. General movement in the open-field activity test
(A) Total distance traveled, (B) total time of movement, and (C) total number of rears during the 30-min open-field test period are shown for all compounds. The 4 doses at which the test compounds were used are represented in black: 0 (vehicle); white: low dose; light grey: medium dose; and dark grey: high dose. The specific doses for each compound (in mg/kg) are indicated in each graph next to the test compound name on the x-axis. N = 4 mice. Data are presented as mean ± S.D.

## Slide 4
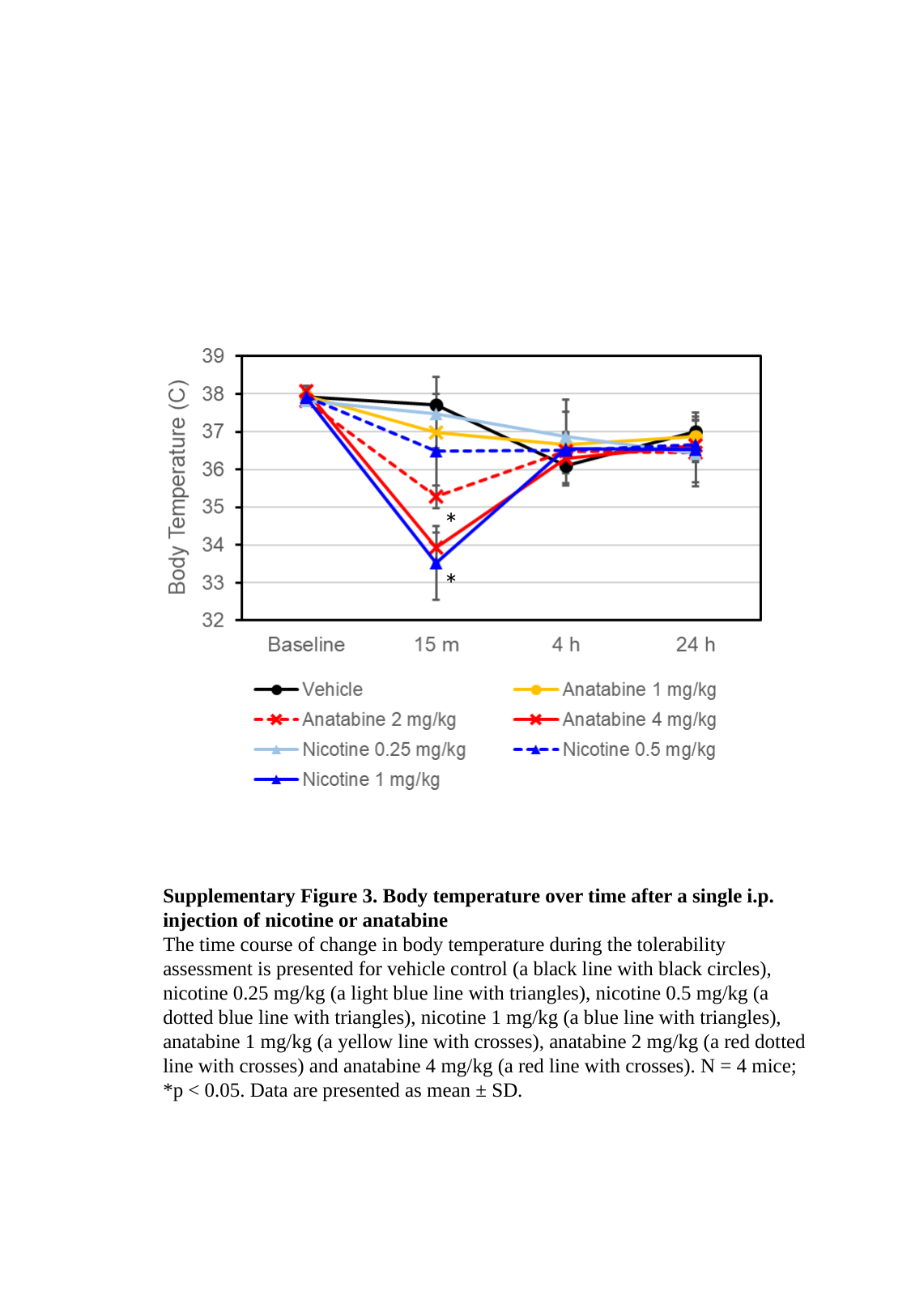

*
*
Supplementary Figure 3. Body temperature over time after a single i.p. injection of nicotine or anatabine
The time course of change in body temperature during the tolerability assessment is presented for vehicle control (a black line with black circles), nicotine 0.25 mg/kg (a light blue line with triangles), nicotine 0.5 mg/kg (a dotted blue line with triangles), nicotine 1 mg/kg (a blue line with triangles), anatabine 1 mg/kg (a yellow line with crosses), anatabine 2 mg/kg (a red dotted line with crosses) and anatabine 4 mg/kg (a red line with crosses). N = 4 mice; *p < 0.05. Data are presented as mean ± SD.

## Slide 5
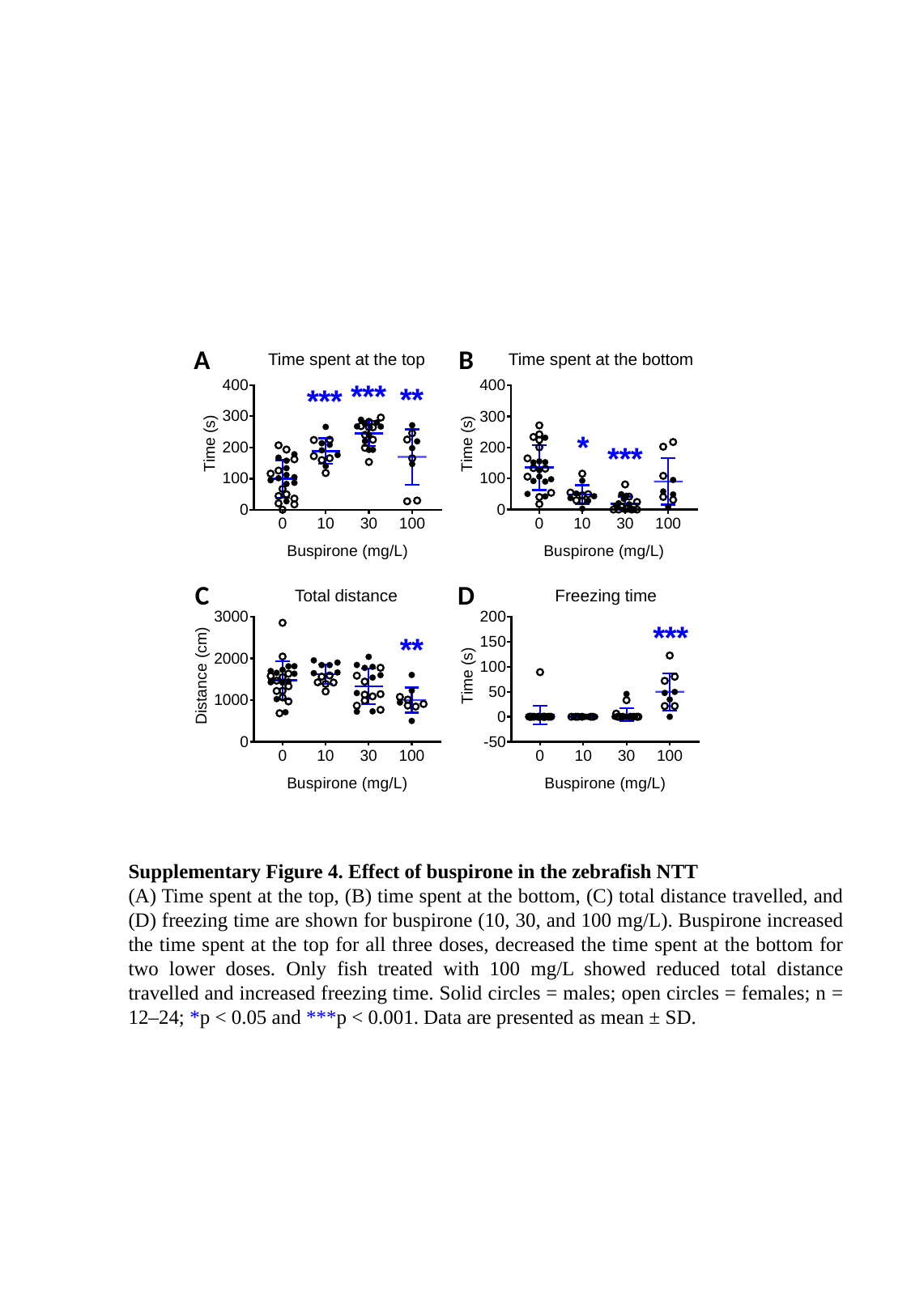

A
B
Time spent at the top
Time spent at the bottom
C
D
Total distance
Freezing time
Supplementary Figure 4. Effect of buspirone in the zebrafish NTT
(A) Time spent at the top, (B) time spent at the bottom, (C) total distance travelled, and (D) freezing time are shown for buspirone (10, 30, and 100 mg/L). Buspirone increased the time spent at the top for all three doses, decreased the time spent at the bottom for two lower doses. Only fish treated with 100 mg/L showed reduced total distance travelled and increased freezing time. Solid circles = males; open circles = females; n = 12–24; *p < 0.05 and ***p < 0.001. Data are presented as mean ± SD.

## Slide 6
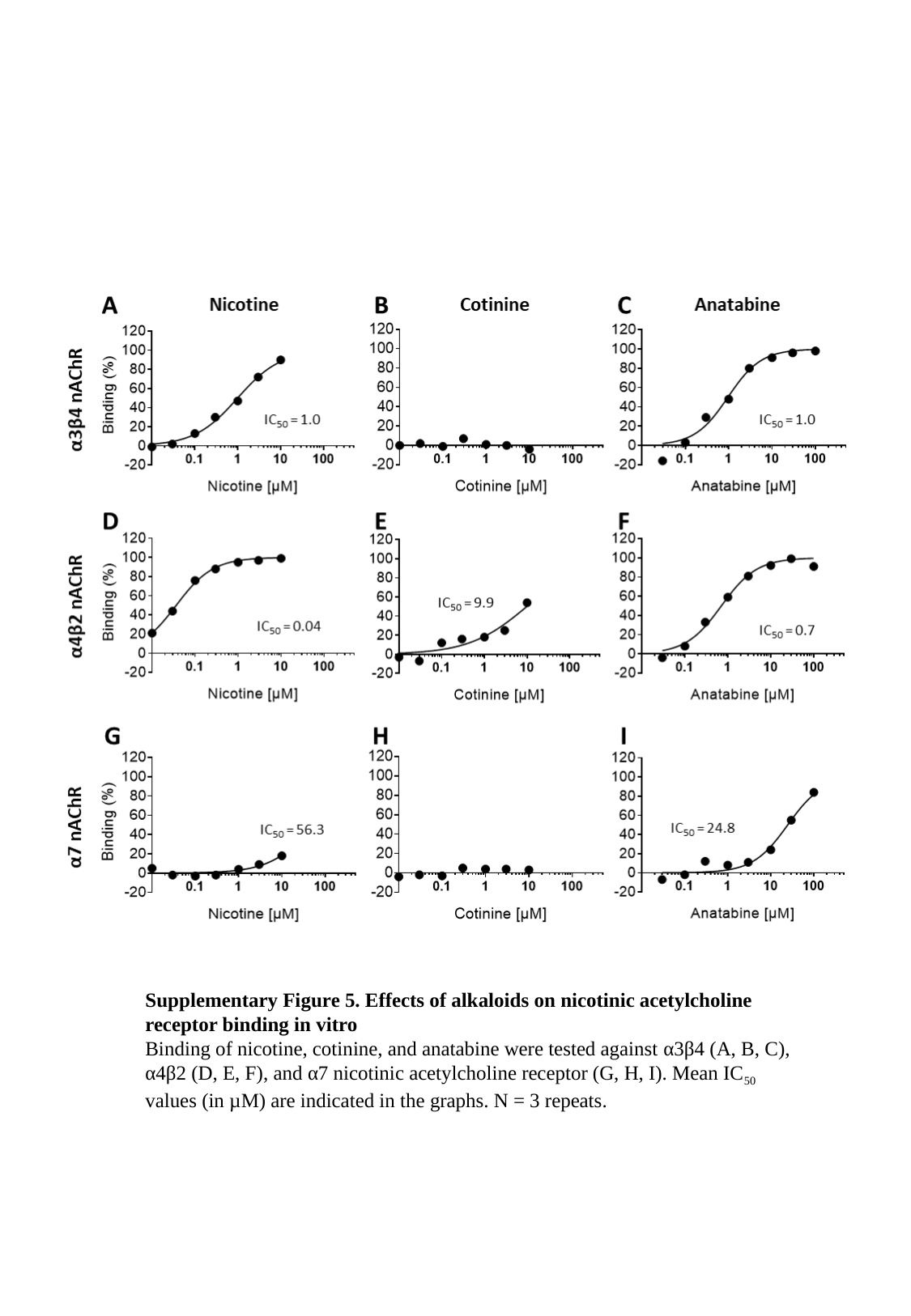

Supplementary Figure 5. Effects of alkaloids on nicotinic acetylcholine receptor binding in vitro
Binding of nicotine, cotinine, and anatabine were tested against α3β4 (A, B, C), α4β2 (D, E, F), and α7 nicotinic acetylcholine receptor (G, H, I). Mean IC50 values (in µM) are indicated in the graphs. N = 3 repeats.
